# Comparative transcriptome and histological analyses of wheat in response to phytotoxic aphid *Schizaphis graminum* and non-phytotoxic aphid *Sitobion avenae* feeding

**DOI:** 10.1186/s12870-019-2148-5

**Published:** 2019-12-10

**Authors:** Yong Zhang, Yu Fu, Jia Fan, Qian Li, Frédéric Francis, Julian Chen

**Affiliations:** 1 State Key Laboratory for Biology of Plant Diseases and Insect Pests, Institute of Plant Protection, Chinese Academy of Agricultural Sciences, Beijing, 100193 People’s Republic of China; 2 Functional and Evolutionary Entomology, Gembloux Agro-Bio Tech, University of Liège, B-5030 Gembloux, Belgium

**Keywords:** *Schizaphis graminum*, *Sitobion avenae*, Wheat, Transcriptome, Cytological, Defense responses, Reactive oxygen species scavengers, Hydrogen peroxide, Chlorosis

## Abstract

**Background:**

Infestation of the phytotoxic aphid *Schizaphis graminum* can rapidly induce leaf chlorosis in susceptible plants, but this effect is not observed with the nonphytotoxic aphid *Sitobion avenae*. However, few studies have attempted to identify the different defence responses induced in wheat by *S. graminum* and *S. avenae* feeding and the mechanisms underlying the activation of chlorosis by *S. graminum* feeding.

**Results:**

*S. graminum* feeding significantly reduced the chlorophyll content of wheat leaves, and these effects were not observed with *S. avenae*. A transcriptomic analysis showed that the expression levels of genes involved in the salicylic acid, jasmonic acid and ethylene signalling defence pathways were significantly upregulated by both *S. avenae* and *S. graminum* feeding; however, more plant defence genes were activated by *S. graminum* feeding than *S. avenae* feeding. The transcript levels of genes encoding cell wall-modifying proteins were significantly increased after *S. graminum* feeding, but only a few of these genes were induced by *S. avenae*. Furthermore, various *reactive oxygen species*-*scavenging gene*s, such as 66 *peroxidase* (*POD*) and 8 *ascorbate peroxidase* (*APx*) genes, were significantly upregulated after *S. graminum* feeding, whereas only 15 *POD* and one *APx* genes were induced by *S. avenae* feeding. The activity of four antioxidant enzymes was also significantly upregulated by *S. graminum* feeding. Cytological examination showed that *S. graminum* feeding induced substantial hydrogen peroxide (H_2_O_2_) accumulation in wheat leaves. The chlorosis symptoms and the loss of chlorophyll observed in wheat leaves after *S. graminum* feeding were reduced and inhibited by the scavenging of H_2_O_2_ by dimethylthiourea, which indicated that H_2_O_2_ plays important role in the induction of chlorosis by *S. graminum* feeding.

**Conclusions:**

*S. graminum* and *S. avenae* feeding induces the JA, SA and ET signalling pathways, but *S. graminum* activated stronger plant defence responses than *S. avenae*. *S. graminum* feeding triggers strong *ROS*-*scavenging activity* and massive H_2_O_2_ production in wheat leaves, and the accumulation of H_2_O_2_ induced by *S. graminum* feeding is involved in the activation of chlorosis in wheat leaves. These results enhance our understanding of mechanisms underlying aphid-wheat interactions and provide clues for the development of aphid-resistant wheat varieties.

## Background

Through interactions with insects over a hundred million years, plants have evolved complex and accurate defence mechanisms against herbivores. In response to herbivory, plants can perceive damage-associated molecular patterns (DAMPs) or herbivory-associated molecular patterns (HAMPs) in insect oral secretions and subsequently induce direct and indirect plant defence responses [1, 2]. Direct defences involve the production and accumulation of plant defensive chemicals, such as plant secondary metabolites (PSMs), proteinase inhibitors, polyphenol oxidases and other defensive proteins, which are induced by herbivory and reduce herbivore performance [3, 4]. Indirect defences include the synthesis and release of complex blends of volatiles that attract parasitoids and predators of the herbivores [5, 6].

Insects from different feeding guilds tend to elicit distinct plant defensive strategies of plants [7, 8]. For example, leaf-eating beetles (Coleoptera) or caterpillars (Lepidoptera) cause extensive tissue damage during herbivory, which usually activates the jasmonic acid (JA)-mediated defence pathway in plants [9, 10]. Different from leaf chewing insects, hemipterans, such as aphids and whiteflies, have highly modified piecing-sucking mouthparts (stylets) that can penetrate the extracellular pathway and feed on the nutrients from phloem sap provided by sieve elements (SEs). Although the stylets puncture through most parts of plant cells during the probing track, these insects cause less damage to cells than leaf-chewing herbivores [11]. Many studies have demonstrated that hemipteran feeding results in the induction of the salicylic acid (SA)-dependent defence pathway in plants [12, 13].

The grain aphid *Sitobion avenae* and the greenbug *Schizaphis graminum* (Hemiptera: Aphididae) are considered two important pests of wheat and other cereals worldwide, as they suck phloem sap and serve as vectors of the barley yellow dwarf virus (BYDV), resulting in significant yield losses [14, 15]. Aphids are classified as nonphytotoxic or phytotoxic according to the symptoms and damage caused by their feeding [16]. Similar to most aphid species, *S. avenae* are nonphytotoxic, and no typical symptoms of plant damage are rapidly induced by their feeding processes. However, infestation by *S. graminum*, a phytotoxic species, can immediately induce obvious leaf chlorosis in susceptible plants, resulting in the deterioration of plant quality and even in plant death [17, 18]. Several studies have characterized the plant defence responses induced by infestation of *S. avenae* and *S. graminum*. For example, Zhao et al. found that *S. avenae* feeding induces the expression of several genes involved in both the SA- and JA-mediated defence pathways in wheat [19], and Zhu-Salzman et al. demonstrated that *S. graminum* feeding strongly induces the expression of genes involved in the SA-dependent pathway in sorghum (*Sorghum bicolor*) but weakly increases the expression levels of JA-related defence genes, such as lipoxidase (*LOX)* and proteinase inhibitors (*PIs*) [20]. Using a cDNA microarray, several transcripts with significantly different expression patterns were identified in sorghum seedlings after *S. graminum* feeding, and these herbivore-responsive genes were mainly associated with photosynthesis, the biosynthesis of defence molecules, cell wall fortification, oxidative bursts and stress [21].

Previous studies have also shown that *S. graminum* feeding significantly increases the concentrations of amino acids, particularly essential amino acids, in the phloem of wheat and barley, and the enhancement of the nutritional quality of plants induced by *S. graminum* feeding improves the aphid performance [22]. It has been proposed that leaf-senescence-like changes triggered by *S. graminum* feeding are associated with the nutritional enhancement of host plants [18, 23]. Feeding damage caused by phytotoxic aphids is usually observed in susceptible hosts; therefore, it has been hypothesized that the nutritional enhancement of plants derived from senescence-like feeding damage is a strategy used by phytotoxic aphids to potentially counteract the negative effects of induced plant resistance, which would eventually improve aphid fitness on the host [18].

However, few studies have attempted to identify the molecular basis of the necrosis symptoms in wheat triggered by the phytotoxic aphid *S. graminum* and the different defence responses induced by *S. graminum* and *S. avenae* feeding. In this study, we integrated gene expression profiling through high-throughput RNA sequencing (RNA-Seq) with cytological examination to reveal the responses of wheat leaves to *S. graminum* and *S. avenae* feeding, to compare the differences in the metabolic pathways affected by the two cereal aphids and to uncover the mechanism underlying the induction of damage symptoms by *S. graminum*.

## Results

### Damage symptoms and changes in the chlorophyll content of wheat leaves after *S. avenae* and *S. graminum* feeding

**Fig. 1:**
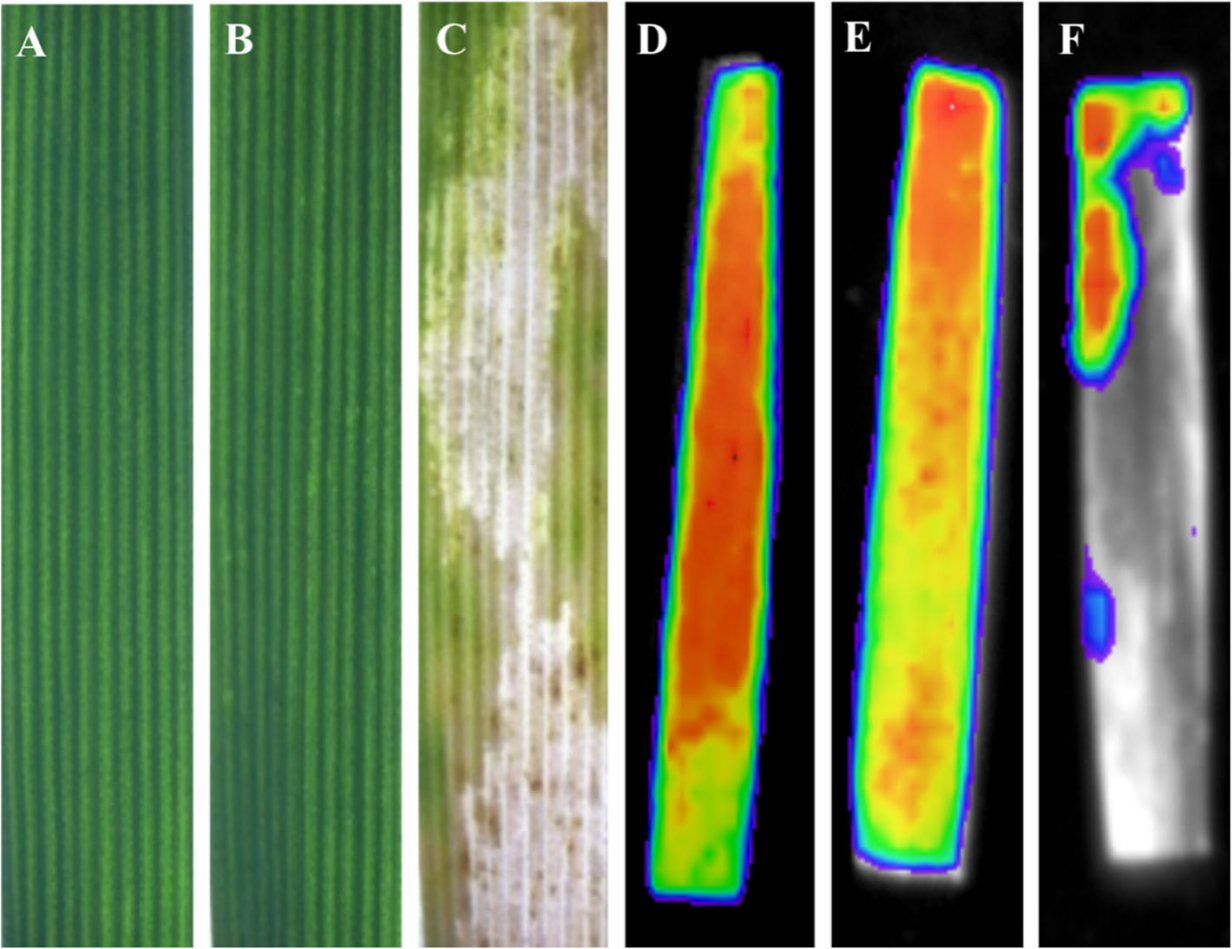
Detection of damage symptoms (**a-c**) and delayed fluorescence (**d-f**) of wheat leaves after aphid infestation. Delayed fluorescence was detected using NightShade LB 985 In vivo Plant Imaging System. Experiments were performed with three biological replicates with similar results, and representative results from one replicate are shown. Red color indicates high intensities representing high chlorophyll content, blue color indicated low intensities of fluorescence, indicating low amounts of chlorophyll. No delayed fluorescence indicates destroyed chlorophyll. **a** and **d**: untreated leaves; **b** and **e** leaves infested with *S. avenae* for 48 h; **c** and **f**: leaves infested with *S. graminum* for 48 h

As shown in Fig. 1a-c, no obvious damage symptoms were detected in leaves 48 h after *S. avenae* infestation compared with leaves without aphid infestation, whereas *S. graminum* feeding caused severe chlorosis in wheat leaves. Delayed fluorescence, which was used as a direct indicator of the chlorophyll content, was also measured in the wheat leaves 48 h after aphid infestation. As demonstrated in Fig. 1d-f, the untreated leaves and the *S. avenae*-infested leaves exhibited strong signals of delayed fluorescence, whereas low signals of delayed fluorescence were detected in the wheat leaves infested with *S. graminum*, which suggested the occurrence of chlorophyll degradation.

**Fig. 2:**
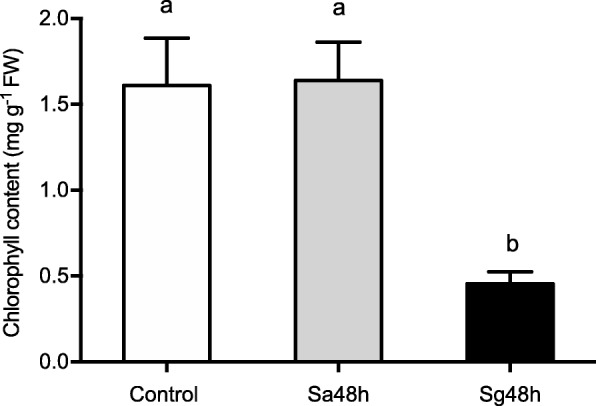
Chlorophyll content of wheat leaves after aphid infestation. Control: untreated leaves; Sa48h: leaves infested with *S. avenae* for 48 h; Sg48h: leaves infested with *S. graminum* for 48 h. The values are presented as means ± SE of three biological replicates. Different letters indicate significant differences among treatments (*P* < 0.05, ANOVA)

The chlorophyll content of the leaves after aphid feeding was further investigated. As shown in Fig. 2, no significant differences in the chlorophyll content were found between the *S. avenae*-infested and control plants. However, the chlorophyll content was significantly decreased to 0.46 ± 0.068 (F_2,6_ = 10.494, *P* = 0.011) after *S. graminum* feeding.

### Transcriptome data from aphid-infested wheat leaves

The transcriptomes of wheat leaves infested with the two cereal aphid species were compared in this study. A total of 62.98 Gb of clean data were obtained from the nine leaf samples, and each of these samples contained ≥6.91 Gb with Q30 quality scores of ≥94.82% (Additional file 1: Table S1). Subsequently, 83.5 to 94.3% of the clean reads from each sample were aligned onto the wheat reference genome and matched to either unique or multiple genomic locations (Table 1).

**Table 1:** Mapping statistics of transcriptome database. The number in brackets indicates the percentage of total reads mapping to the wheat reference genome and/or matched at either multiple or unique genomic loci

Sample name	Total reads	Total mapped	Multiple mapped	Uniquely mapped
Control-1	46,272,278	42,946,912 (92.81%)	4,147,714 (8.96%)	38,799,198 (83.85%)
Control-2	46,669,950	43,353,788 (92.89%)	4,598,942 (9.85%)	38,754,846 (83.04%)
Control-3	47,129,428	44,452,968 (94.32%)	4,238,840 (8.99%)	40,214,128 (85.33%)
Sa48h-1	46,058,314	43,100,126 (93.58%)	3,812,164 (8.28%)	39,287,962 (85.3%)
Sa48h-2	46,068,740	42,785,254 (92.87%)	3,721,176 (8.08%)	39,064,078 (84.8%)
Sa48h-3	46,611,206	43,536,070 (93.4%)	3,934,960 (8.44%)	39,601,110 (84.96%)
Sg48h-1	46,754,444	39,079,032 (83.58%)	3,388,782 (7.25%)	35,690,250 (76.34%)
Sg48h-2	47,294,858	43,947,242 (92.92%)	3,980,232 (8.42%)	39,967,010 (84.51%)
Sg48h-3	47,136,410	43,851,752 (93.03%)	3,760,518 (7.98%)	40,091,234 (85.05%)

### Identification and functional annotation of DEGs

**Fig. 3:**
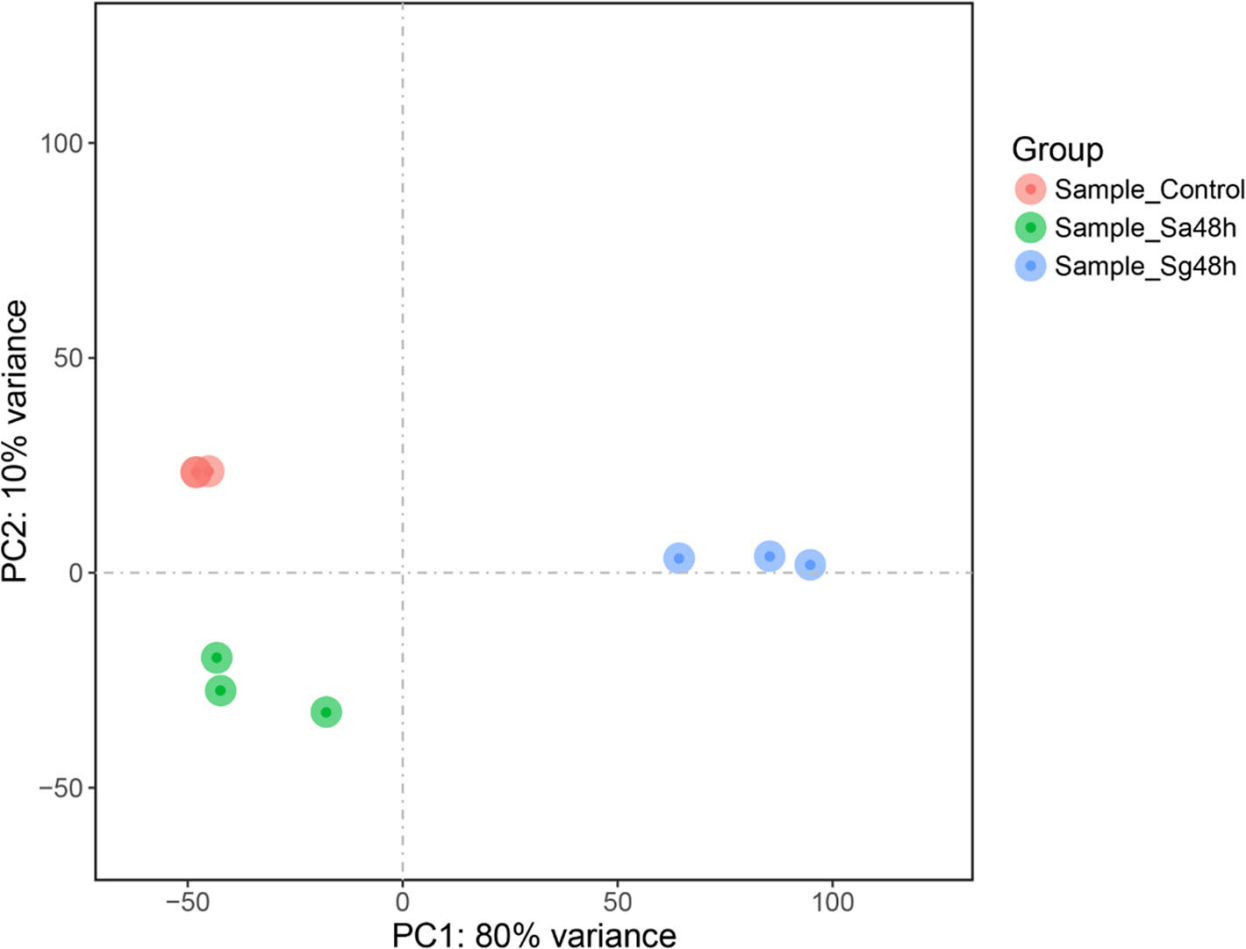
Principal component analysis (PCA) plot on transcriptome data from control groups (red spots), wheat leaves infested with *S. avenae* for 48 h (Sa48h, green spots) and wheat leaves infested with *S. graminum* for 48 h (Sg48h, blue spots)

**Fig. 4:**
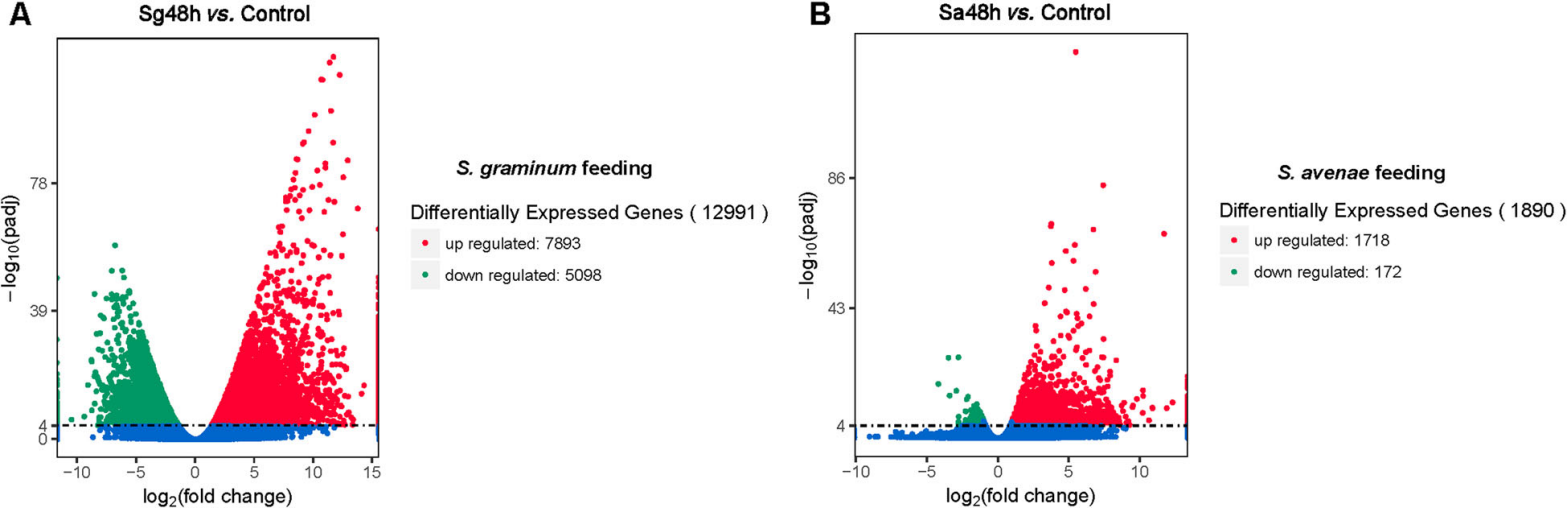
Volcano plots of differentially expressed genes (DEGs) between the aphid-free wheat leaves and those infested with *S. graminum* (**a**) or *S. avenae* (**b**) for 48 h. Each dot represents one gene with the y-axis showing -log_10_ *q* value and the x-axis showing log_2_ fold change, respectively. The red, green and blue dots represent the up-regulated DEGs, down-regulated DEGs (adjusted *p* values < 0.00001, |log_2_ FC| ≥ 1) and not significantly changed genes, respectively

The gene expression levels of each replicate were assessed through principal component analysis (PCA). Replicates from the same group were clustered closely together, which suggested that the repeatability of each treatment group was satisfactory, and the samples from the *S. avenae*- and *S. graminum*-infested groups (Sa48h and Sg48h, respectively) clustered far from the control samples, which indicated that aphid feeding induced significant changes in gene expression (Fig. 3). A total of 12,8195 transcripts were detected across all the samples (Additional file 2: Data S1). Gene expression levels with an adjusted *P* value < 0.00001 and |Log_2_Fold Change| ≥ 1 were selected as DEGs for further analysis. Forty-eight hours of *S. avenae* feeding significantly upregulated 1718 genes and significantly downregulated 172 genes in wheat leaves (Fig. 4a). In addition, 7893 and 5098 genes were significantly upregulated and downregulated, respectively, after 48 h of *S. graminum* feeding (Fig. 4b).

**Fig. 5:**
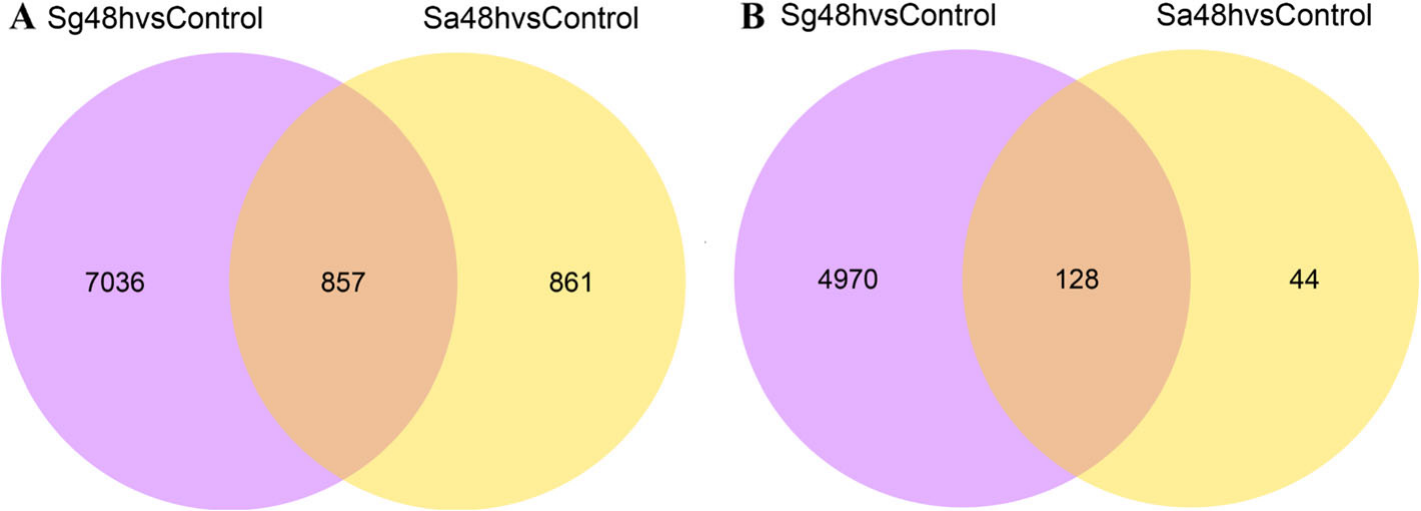
Venn diagram of DEGs with upregulation (**a**) and downregulation (**b**) patterns after *S. graminum* (Sg48h) and *S. avenae* (Sa48h) feeding. Overlapping parts represent genes significantly regulated by both *S. graminum* and *S. avenae* feeding

To investigate the differences in plant responses to infestation by *S. graminum* and *S. avenae*, the DEGs in wheat leaves induced by these aphids were also compared in our study. The results showed that the expression levels of 857 genes in wheat leaves were significantly upregulated by both *S. graminum* and *S. avenae* feeding. Additionally, 11,046 and 1914 transcripts were specifically and significantly upregulated after *S. graminum* and *S. avenae* feeding for 48 h, respectively (Fig. 5a). In contrast, a total of 128 transcripts were significantly downregulated after both *S. graminum* and *S. avenae* infestation, and 7036 and 861 genes were only significantly downregulated after *S. graminum* and *S. avenae* feeding, respectively (Fig. 5b). This finding suggested that the global response of wheat to *S. graminum* feeding is distinct from that of wheat to *S. avenae* feeding.

**Fig. 6:**
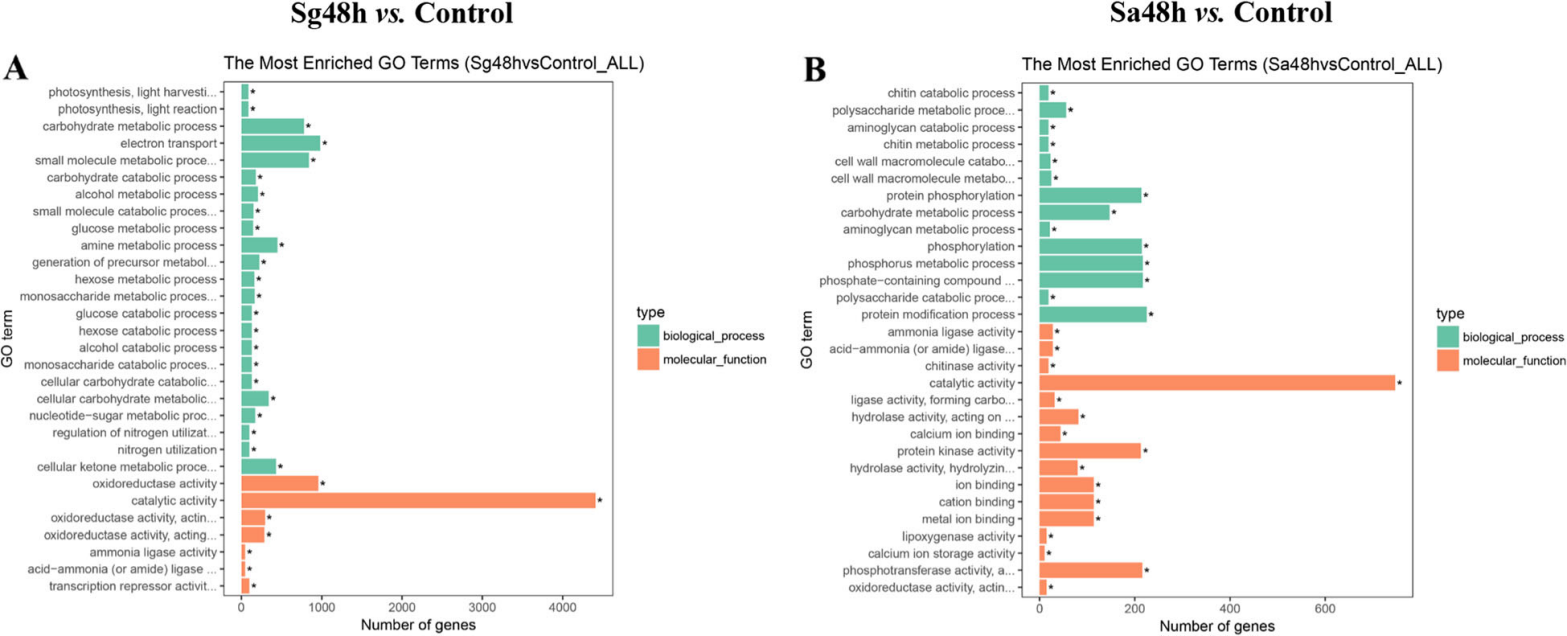
GO enrichment analysis of the DEGs in wheat leaves infested with *S. graminum* (**a**) and *S. avenae* (**b**) for 48 h. Control: untreated leaves; Sg48h: leaves infested with *S. graminum* for 48 h; Sa48h: leaves infested with *S. avenae* for 48 h

All the DEGs were subjected to GO term and KEGG pathway analyses to identify the major DEG-associated metabolic pathways. The top 30 enriched GO terms and 20 most enriched KEGG pathways are shown in Fig. 6 and Figure S1 (Additional file 3). As shown in Fig. 6, within the biological process category, the DEGs induced by *S. graminum* were mainly enriched in the electron transport, small molecule metabolic process and carbohydrate metabolic process terms, and the DEGs induced by *S. avenae* were mainly enriched in protein phosphorylation, protein modification process and phosphorus metabolic process. Within the molecular function category, the largest proportion of DEGs induced by *S. graminum* was enriched in the catalytic activity and oxidoreductase activity terms, and the majority of the DEGs activated by *S. avenae* were enriched in the catalytic activity, protein kinase activity and phosphotransferase activity.

### Transcripts related to photosynthesis, sucrose and starch metabolism and nitrogen metabolism

*S. graminum* feeding negatively affected the photosynthesis process of wheat, and many genes associated with light-harvesting and photosystem-associated genes, such as chlorophyll a-b binding proteins, ferrochelatase, and photosystem I and II proteins, were significantly downregulated (Table 2). The expression levels of *ribulose bisphosphate carboxylase oxygenase* (*RuBisCO*) and *carbonic anhydrase* genes with roles in the Calvin cycle were also significantly reduced after *S. graminum* feeding. However, few genes involved in photosynthesis were significantly regulated in the *S. avenae*-infested plants. The transcriptional profiles of some genes involved in sucrose and starch metabolism were also investigated. The *sucrose synthase 3* gene, one *trehalose-6-phosphate synthase* gene and six *beta-glucosidase* genes were significantly downregulated in *S. graminum-* and *S. avenae*-infested leaves, and the transcript levels of *sucrose-phosphatase* genes were significantly upregulated in wheat leaves infested with *S. graminum* and *S. avenae*. *S. graminum* feeding but not *S. avenae* feeding also significantly affected nitrogen metabolism. The transcript levels of *nitrate reductase* in the leaves were strongly downregulated by *S. graminum*, and *glutamate dehydrogenase* was significantly upregulated in wheat leaves infested with *S. graminum*. However, few genes involved in nitrogen metabolism were modulated by *S. avenae* feeding.

**Table 2:** DEGs associated with primary plant metabolism in wheat leaves in response to *S. graminum* (Sg48h) and *S. avenae* (Sa48h) feeding

Pathways	Gene description	Gene ID	Sg 48 h		Sa 48 h	
			Log_2_ FC	*q*-value	Log_2_ FC	*q*-value
photosynthesis	Chlorophyll a-b binding protein	TraesCS5B02G462800	−8.2468	4.71E-05	/	
		TraesCS5A02G454200	−7.2799	4.32E-10		
		TraesCS5B02G462900	−7.4537	1.36E-05		
		TraesCS1D02G411600	−7.5869	1.42E-09		
		TraesCS1A02G403800	−7.1482	3.43E-28		
		TraesCS6A02G094200	−7.2817	3.20E-16		
		TraesCS6A02G094500	−7.038	3.66E-08		
		TraesCS6D02G088800	−6.9431	5.03E-06		
		TraesCS5D02G464700	−6.8985	5.19E-11		
		TraesCS5B02G463000	−6.8386	3.60E-09		
		TraesCS6A02G094600	−6.8352	1.98E-05		
	Ferrochelatase	TraesCS1A02G135700	−1.6923	4.67E-06	/	
		TraesCS1D02G131200	− 1.9256	1.97E-07		
	Photosystem I proteins	TraesCS2D02G253200	−4.3659	6.19E-31	/	
		TraesCS1B02G420100	−3.9963	2.67E-25		
		TraesCS2A02G252600	−3.9493	2.05E-26		
		TraesCS2B02G272300	−3.8273	3.85E-25		
		TraesCS1A02G392000	−3.7654	3.25E-24		
		TraesCS5A02G256900	−3.7814	2.36E-24		
	Photosystem II proteins	TraesCS4A02G355600	−3.9717	4.97E-07	/	
		TraesCS5A02G386400	−3.6099	6.97E-11		
		TraesCS7B02G215000	−3.6384	2.27E-22		
		TraesCS7A02G314100	−2.8644	3.79E-15		
		TraesCS3B02G344200	−2.9397	4.08E-16		
		TraesCS7A02G314100	−3.1573	5.97E-18		
	Photosynthetic NDH	TraesCS6D02G287300	−3.8223	1.11E-18	/	
		TraesCS6A02G308100	−4.9624	1.33E-19		
		TraesCS4D02G273000	−3.3857	1.73E-17		
	Carbonic anhydrase	TraesCS3A02G230000	−6.5051	1.82E-15	/	
		TraesCS3B02G259300	−6.7942	9.39E-14		
		TraesCS3D02G223300	−6.4226	4.53E-12		
	RuBisCO	TraesCS2D02G065400	−7.0835	1.64E-06	/	
		TraesCS2D02G065300	−6.3924	6.01E-08		
		TraesCS2D02G065100	−6.1885	6.39E-06		
		TraesCS2D02G065200	−5.3796	6.33E-06		
sucrose and starch metabolisms	Trehalose-6-phosphate synthase	TraesCS1A02G339300	−2.2863	1.92E-09	−1.1024	6.64E-05
	sucrose synthase 3	TraesCS2A02G168200	−3.4137	1.60E-09	−2.3629	7.91E-05
	Sucrose-phosphatase	TraesCS1B02G107600	4.0937	4.86E-10	3.0633	8.93E-09
	Beta-glucosidase	TraesCS2B02G401500	−2.1186	1.62E-09	−1.602	3.62E-07
		TraesCS2D02G381000	−2.1015	1.30E-09	−1.4152	3.85E-10
		TraesCS3D02G440200	−6.7792	7.04E-60	−1.0065	2.14E-06
nitrogen metabolisms	Nitrate reductase	TraesCS6B02G356800	Induced	6.33E-16	/	
		TraesCS6D02G306000	Induced	9.79E-17		
		TraesCS6A02G326200	Induced	3.11E-10		
	Glutamine synthetase	TraesCS2A02G500400	−3.0056	8.28E-07	/	
		TraesCS2B02G528300	−3.0711	6.95E-09		
		TraesCS2D02G500600	−3.7958	5.12E-24		
		TraesCS6B02G327500	1.9819	1.68E-08		
	Glutamate dehydrogenase	TraesCS2B02G409300	6.9374	3.09E-08	/	
		TraesCS2A02G389900	6.5323	1.14E-06		
	Cysteine synthase	TraesCS3A02G338600	−2.8015	5.21E-06	/	
		TraesCS3B02G370200	−2.9708	1.56E-05		
		TraesCS4A02G401600	−2.4908	3.85E-09		
		TraesCS6B02G217200	3.9529	2.04E-07		

“/” indicates no significant differences between aphid infested and control groups

### Effects of *S. graminum* and *S. avenae* feeding on transcripts related to the SA, JA, and ET signalling pathways involved in plant defence

To characterize how plant defence responses are modulated in response to *S. graminum* and *S. avenae* feeding, genes known to be involved in the SA, JA, and ET-defence pathways were examined [24]. The transcriptome data in Table 3 showed that 21 *PAL* genes involved in SA biosynthesis were significantly upregulated in response to *S. graminum* and *S. avenae* feeding. Furthermore, 13 *PR* genes responding to SA were significantly upregulated by *S. graminum* and *S. avenae* feeding. Additionally, under *S. graminum* and *S. avenae* feeding, one *AOC* (3.7-fold), three *AOS* (2.7- to 7.9-fold) and five *LOX* (2.8- to 8.8-fold) genes involved in JA biosynthesis were significantly upregulated, and the expression levels of three *PI* genes, which are JA-responsive defence genes, significantly increased. Two *ACS* (5.1 to 7.8-fold) and three *ACO* (4.0 to 5.0-fold) genes, which are involved in the ET signalling pathway, and 11 genes encoding ethylene-responsive transcription factors involved in ET biosynthesis were significantly upregulated in response to *S. graminum* feeding, but only one *ACO* (4.4-fold) gene and two genes encoding ethylene-responsive transcription factors (1.8-fold and induced) were upregulated after *S. avenae* feeding.

**Table 3:** DEGs involved in SA, JA and ET- dependent defense pathways in response to *S. graminum* (Sg48h) and *S. avenae* (Sa48h) feeding

Pathways	Gene description	Gene ID	Sg 48 h		Sa 48 h	
			Log_2_ FC	*q*-value	Log_2_ FC	*q*-value
SA-defense pathway	Phenylalanine ammonia-lyase (PAL)	TraesCS1A02G037700	Induced	4.21E-06	Induced	1.22E-06
		TraesCS1B02G122800	6.71	7.58E-09	6.98	1.38E-06
		TraesCS1D02G039300	6.76	2.51E-23	7.13	4.31E-06
		TraesCS1D02G039400	6.45	3.28E-07	7.09	7.79E-07
		TraesCS1D02G103500	5.75	1.54E-05	5.88	6.89E-07
		TraesCS2A02G196400	5.33	1.18E-05	5.17	1.62E-06
		TraesCS2A02G196700	Induced	3.75E-20	Induced	2.47E-07
		TraesCS2A02G381000	6.24	2.33E-11	5.90	2.08E-06
		TraesCS2A02G381100	5.14	2.20E-16	4.33	3.11E-05
		TraesCS2B02G224300	7.90	9.99E-09	8.22	2.83E-07
		TraesCS2B02G398100	3.39	2.81E-05	4.57	7.02E-12
		TraesCS2B02G398200	5.80	2.59E-17	5.04	6.19E-06
		TraesCS2B02G398400	4.79	6.14E-10	4.80	1.40E-05
		TraesCS2D02G204400	9.53	4.40E-07	8.44	3.35E-07
		TraesCS2D02G377200	4.54	7.91E-23	4.32	4.15E-07
		TraesCS2D02G377500	5.51	4.27E-26	4.43	5.23E-05
		TraesCS4A02G401300	9.34	2.75E-13	9.48	9.50E-11
		TraesCS5B02G468300	5.52	2.74E-09	7.64	2.18E-11
		TraesCS5B02G468400	5.24	5.32E-06	6.35	1.20E-07
		TraesCS6A02G222700	4.72	8.97E-06	6.48	1.28E-14
		TraesCS6B02G258600	6.08	8.58E-11	5.77	5.11E-07
	Pathogenesis-related protein (PR protein)	TraesCS1A02G355300	7.37	1.52E-65	5.43	1.90E-64
		TraesCS1B02G366300	Induced	1.05E-15	Induced	8.39E-05
		TraesCS2D02G317800	7.69	1.16E-15	8.12	4.17E-09
		TraesCS3A02G480400	10.19	5.99E-14	5.34	1.02E-12
		TraesCS3B02G525000	10.30	1.40E-12	6.20	5.96E-29
		TraesCS3D02G475200	8.93	4.04E-15	5.55	3.58E-06
		TraesCS4D02G015800	7.02	6.75E-26	3.27	3.39E-05
		TraesCS5A02G018200	5.72	7.89E-17	4.26	4.95E-26
		TraesCS5A02G183300	9.92	7.93E-60	5.56	1.27E-30
		TraesCS5A02G439800	12.67	7.78E-14	7.21	1.44–09
		TraesCS5B02G181500	9.93	1.35E-77	5.67	1.11E-37
		TraesCS5B02G442700	13.32	4.11E-05	7.26	3.37E-06
		TraesCS7D02G161200	10.06	2.20E-20	6.19	5.78E-50
JA-defense pathway	Allene oxide synthase (AOS)	TraesCS4A02G061900	7.09	3.88E-13	6.96	5.85E-08
		TraesCS4B02G237600	5.54	2.23E-06	6.23	6.93E-17
		TraesCS4D02G238800	2.77	1.19E-12	4.42	2.27E-08
	Allene oxide cyclase (AOC)	TraesCS6D02G314300	3.70	5.68E-05	2.06	2.39E-05
	Lipoxygenase (LOX)	TraesCS4B02G037700	2.84	8.85E-15	3.44	2.11E-09
		TraesCS4B02G037800	3.75	2.55E-22	2.79	2.17E-06
		TraesCS4B02G037900	4.70	8.54E-36	4.47	8.67E-08
	Proteinase inhibitors (PIs)	TraesCS3A02G046100	8.32	1.53E-29	4.71	7.90E-09
		TraesCS3B02G038700	8.81	2.09E-21	5.85	1.51E-15
		TraesCS3D02G034200	Induced	2.08E-10	Induced	3.50E-09
ET-signaling pathway	1-aminocyclopropane-1-carboxylate synthase (ACS)	TraesCS2B02G414800	7.84	5.12E-08	/	/
		TraesCS2D02G394200	5.10	3.76-E05		
	1-aminocyclopropane-1-carboxylate oxidase 1 (ACO)	TraesCS6B02G355900	4.43	4.11-E09	/	/
		TraesCS6A02G325700	4.03	6.84E-07		
		TraesCS6B02G356200	5.04	2.31E-11		
		TraesCS4A02G109200	/	/	4.39	8.67E-05
	ET-responsive transcription factors	TraesCS6B02G281000	Induced	1.18E-08	Induced	4.12E-05
		TraesCS4A02G326400	Induced	6.85E-26	/	/
		TraesCS4D02G313400	Induced	2.10E-05		
		TraesCS1B02G282300	Induced	7.57E-05		
		TraesCS2D02G414300	Induced	1.72E-06		
	ET-insensitive protein	TraesCS5A02G547500	−2.1748	1.73E-08	−1.2853	3.07E-07
		TraesCS4A02G275600	−3.3408	1.20E-09	/	/
		TraesCS4D02G036000	−3.5712	4.72E-16		
		TraesCS7B02G145400	−2.3144	1.46E-07		

“/” indicates no significant differences between aphid-infested and control groups

Although the transcript levels of some defence genes were significantly upregulated in response to both *S. graminum* and *S. avenae* feeding, a higher number of DEGs involved in SA-, JA-, and ET-mediated defence pathways were induced by *S. graminum* feeding than by *S. avenae* feeding (Additional file 4: Data S2). For example, 37 *PR* genes (downstream of SA) and 10 *PI* genes (downstream of JA) were significantly upregulated in response to *S. graminum* feeding, but only 17 *PR* genes and four *PI* genes were significantly upregulated in response to *S. avenae* feeding. Additionally, *S. graminum* feeding induced greater fold changes in these two types of genes than *S. avenae* feeding, which indicated that the former triggered a stronger defence response than the latter.

### Effects of *S. graminum* feeding on transcripts associated with plant cell wall modification proteins (PCMDPs) in wheat leaves

*S. graminum* feeding induced the expression of many genes encoding enzymatic or non-enzymatic proteins related to plant cell wall dynamics (Table 4). For example, the transcript levels of callose synthases were significantly upregulated after *S. graminum* and *S. avenae* feeding. The transcript levels of 19 *PGs* (1.92 to 6.45-fold; induced) and four *PEMs* (3.37 to 3.97-fold; induced) were significantly increased after 48 h of *S. graminum* feeding, but no *PGs* or *PEMs* were significantly induced in response to *S. avenae* feeding. Similarly, the transcript levels of six genes encoding beta-expansin, which is a non-enzymatic protein that plays important roles in cell wall loosening, were significantly upregulated (5.30 to 8.70-fold; induced) in wheat leaves infested with *S. graminum*, but no *expansin* genes were significantly regulated after 48 h of *S. avenae* feeding.

**Table 4:** Expression levels of plant cell wall-modifying proteins in response to *S. graminum* (Sg48h) and *S. avenae* (Sa48h) feeding in wheat leaves

Gene description	Gene ID	Sg48h		Sa48h	
		Log_2_ FC	*q* value	Log_2_ FC	*q* value
Callose synthases	TraesCS2D02G600000	2.5134	3.11E-05	3.1012	1.02E-05
	TraesCSU02G021500	3.1218	4.49E-05	4.849	1.25E-12
Pectin acetylesterase (PAE)	TraesCS2A02G377100	7.2447	6.75E-10	/	/
	TraesCS5A02G486000	−1.8876	3.22E-06	/	/
	TraesCS2B02G178100	/	/	2.6875	2.00E-10
	TraesCS2D02G158400	/	/	2.5945	2.76E-08
Polygalacturonase (PG)	TraesCS1A02G311100	3.10	1.01E-14	/	
	TraesCS1A02G364800	6.45	2.20E-05		
	TraesCS1D02G311000	2.20	2.94E-07		
	TraesCS2D02G421700	5.18	5.23E-05		
	TraesCS3B02G258600	3.67	4.23E-05		
	TraesCS3D02G223900	4.70	2.26E-09		
	TraesCS4B02G086500	3.81	8.56E-21		
	TraesCS5A02G212900	2.68	1.42E-08		
	TraesCS5A02G459000	Induced	9.07E-06		
	TraesCS5A02G513600	3.75	3.75E-15		
	TraesCS5B02G210900	2.03	6.33E-06		
	TraesCS5B02G468700	Induced	1.08E-09		
	TraesCS5D02G102900	3.08	4.19E-11		
	TraesCS5D02G219200	2.02	1.29E-06		
	TraesCS5D02G470100	Induced	1.03E-07		
	TraesCS6A02G084500	Induced	2.92E-06		
	TraesCS6B02G118000	Induced	2.39E-05		
	TraesCS7D02G326800	6.27	5.33E-09		
	TraesCS1B02G322500	1.92	8.26E-05		
Pectinesterase (PEM)	Novel04561	3.37	4.08E-09	/	
	TraesCS1A02G263900	Induced	3.93E-38		
	TraesCS1D02G264000	Induced	5.08E-07		
	TraesCS2A02G173200	3.97	1.76E-09		
Beta-expansin (EXP)	TraesCS1A02G212300	7.17	2.96E-10	/	
	TraesCS1B02G225700	−4.59	4.09E-09		
	TraesCS1B02G225900	5.30	6.23E-06		
	TraesCS1D02G215300	6.65	5.54E-13		
	TraesCS3A02G480000	8.70	2.20E-07		
	TraesCS3B02G524500	8.61	1.25E-06		
	TraesCS5A02G073100	Induced	5.03E-08		

“/” indicates no significant differences between aphid-infested and control groups

### Effects of *S. graminum* and *S. avenae* feeding on the transcript levels and activities of antioxidant enzymes involved in ROS scavenging in wheat leaves

**Fig. 7:**
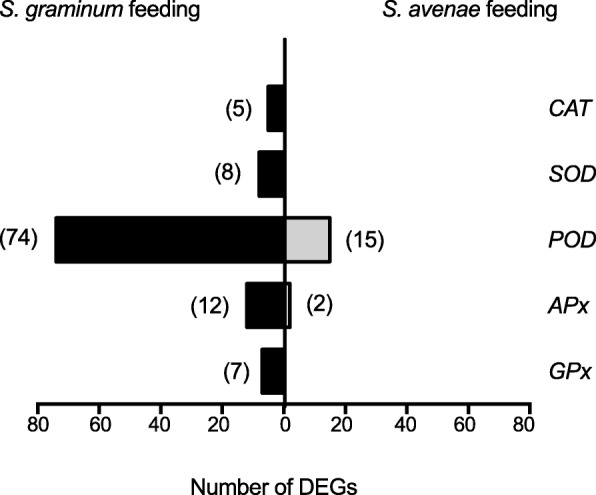
Number of DEGs induced or repressed after 48 h of *S. graminum* (black bars) and *S. avenae* (grey bars) feeding in ROS scavenging enzymes. The total number of characterized genes in each enzyme is shown in parentheses following the bars

In plants, herbivore attacks usually trigger oxidative responses [25]. Plants possess a battery of ROS scavengers, such as POD, SOD, and CAT enzymes, and these enzymes can protect cells from oxidative damage [26]. As shown in Fig. 7, 74 *PODs* were significantly up- or down- regulated in response to *S. graminum* feeding, and 66 of these *PODs* were significantly upregulated. However, only 15 *PODs* were significantly upregulated by *S. avenae* feeding. Similarly, the expression levels of 12 *APx* genes were significantly modulated by *S. graminum* feeding, but the expression levels of only two *APx* genes were significantly affected by *S. avenae* feeding. Additionally, five *CAT*, eight *SOD* and seven *glutathione peroxidase* (*GPx*) genes were significantly regulated by *S. graminum* feeding, but not by *S. avenae* feeding (Additional file 5: Data S3). The increased number of ROS scavengers induced by *S. graminum* feeding suggested that *S. graminum* feeding induces stronger oxidative stress in wheat leaves than *S. avenae*.

**Fig. 8:**
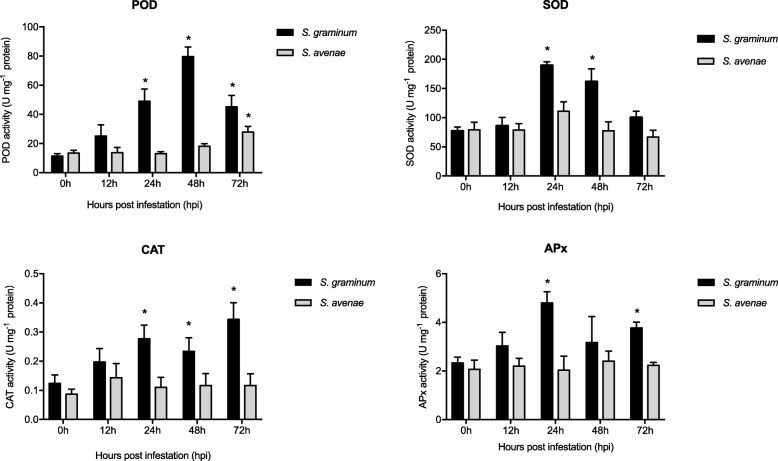
Effects of *S. graminum* and *S. avenae* feeding on the activities of POD, SOD, CAT and APx in wheat leaves at different time points. The values are presented as means ± SE of three biological replicates. Leaves without aphid infestation at 0 h were set as control groups. Asterisks indicate the mean values are significantly different between the aphid-infested and control plants (*P* < 0.05)

Compared with the control, the activity of POD was significantly increased after 24 h (t_4_ = − 4.387, *P* = 0.012) of *S. graminum* feeding and reached a peak at 48 h (t_4_ = − 9.981, *P* = 0.001) (Fig. 8). The activity of POD in leaves was also significantly increased 72 h after *S. avenae* feeding (t_4_ = − 3.353, *P* = 0.028). Furthermore, the activities of SOD, CAT and APx were significantly increased after 24 h of *S. graminum* feeding (t_4_ = − 12.295, *P* < 0.001; t_4_ = − 2.789, *P* = 0.049; t_4_ = − 7.761, *P* = 0.001), whereas *S. avenae* feeding had no significant effects on the activities of SOD, CAT and APx (t_4_ = − 1.560, *P* = 0.194; t_4_ = − 0.600, *P* = 0.581; t_4_ = 0.048, *P* = 0.964).

### Cytological examination of callose deposition and ROS accumulation in aphid-infested leaves

**Fig. 9:**
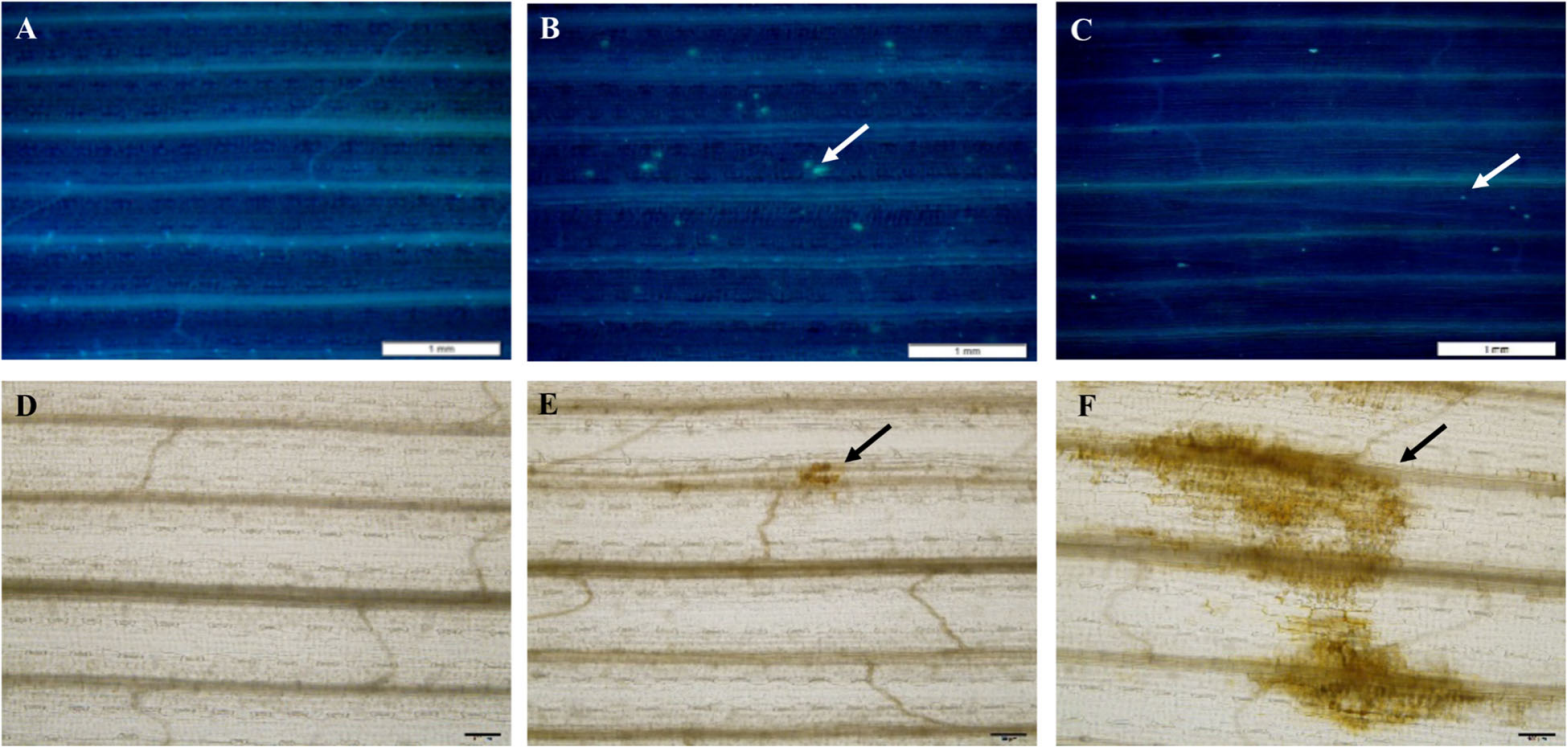
Cytological examination of aphid-infested wheat leaves for H_2_O_2_ accumulation and callose deposition. Experiments were performed with three biological replicates with similar results, and representative results from one replicate are shown. **a-c**: aniline blue staining for callose deposition (scale bar = 1 mm). **d-f**: DAB staining to detect H_2_O_2_ (scale bar = 100 μm). Untreated control leaves (**a** and **d**); *S. avenae*-infested leaves (**b** and **e**); *S. graminum*-infested leaves (**c** and **f**)

To detect whether callose was deposited at the feeding sites, aphid-infested leaves were stained with aniline blue. As shown in Fig. 9, no callose deposits were observed in vascular tissues without aphid infestation (Fig. 9a). However, in *S. avenae-* and *S. graminum*-infested tissues, callose deposits were clearly detected as bright blue fluorescence directly at the feeding sites (Fig. 9b and c).

**Fig. 10:**
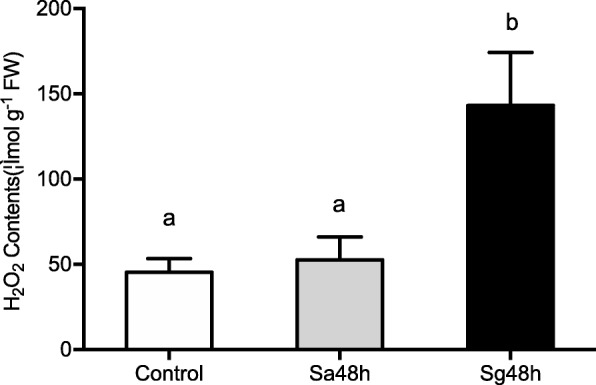
Content of H_2_O_2_ in wheat leaves after infestation by aphids. Control: untreated leaves; Sa48h: 48 h post *S. avenae* feeding; Sg48h: 48 h post *S. graminum* feeding. The values are presented as means ± SE of three biological replicates. Different letters indicate significant differences among treatments (*P* < 0.05, ANOVA)

H_2_O_2_ accumulation has been shown to be induced by wounding and by pathogen and herbivore attacks in plants and is involved in plant defence responses as a signal molecule [27]. To record the accumulation of H_2_O_2_ after aphid infestation, *S. avenae*- and *S. graminum*-infested leaves were examined after cytological staining with DAB, which was used to detect the production of H_2_O_2_. As shown in Fig. 9d-e, no obvious DAB staining was observed in the non-infested leaves, and a small brown-stained area was detected in the wheat leaves after *S. avenae* feeing. However, H_2_O_2_ was clearly detected in the areas of *S. graminum* feeding, which indicated that *S. graminum* feeding induced a massive accumulation of H_2_O_2_ (Fig. 9f). The H_2_O_2_ contents in the feeding sites of wheat leaves after *S. avenae* and *S. graminum* infestations were also examined. As indicated in Fig. 10, the concentration of H_2_O_2_ in wheat leaves infested with *S. graminum* (143.19 ± 31.15 μmol g^− 1^ FW; F_2,6_ = 7.345; *P* = 0.024) was significantly higher than that in *S. avenae*-infested and control leaves. In contrast, *S. avenae* feeding had no significant effects on the H_2_O_2_ content compared with the control (Fig. 10). The changes in the H_2_O_2_ content in response to aphid feeding were consistent with the DAB staining results.

### Scavenging of H_2_O_2_ using DMTU reduces *S. graminum* feeding-induced damage on wheat leaves

**Fig. 11:**
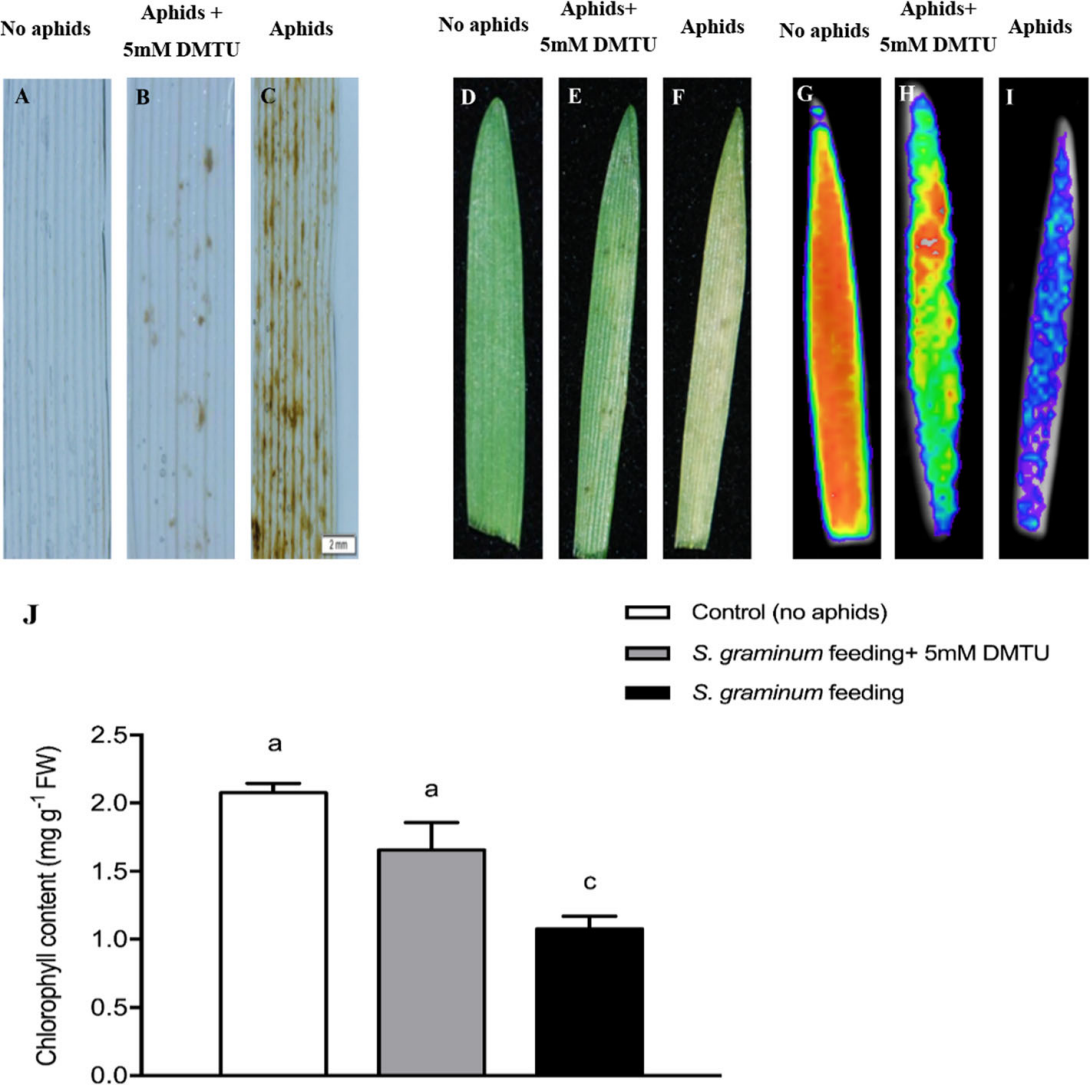
Effects of H_2_O_2_ scavenging by DMTU on feeding damage symptoms and total chlorophyll content induced by *S. graminum* feeding in wheat leaves. **a-c**: DAB staining to detect H_2_O_2_ in wheat leaves (scale bar = 2 mm). **d-i**: feeding damage symptoms and degradation of chlorophyll in wheat leaves. **j**: total chlorophyll content in wheat leaves treated with 5 mM DMTU. Different letters indicate significant differences among treatments (*P* < 0.05, ANOVA). The values are presented as means ± SE of three biological replicates. A, D, G: leaves without aphid infestation; B, E, H: leaves infested with aphids treated with 5 mM DMTU; C, F, I: leaves infested with aphids

To further investigate the role of H_2_O_2_ accumulation on the damage induced by *S. graminum* feeding, wheat seedlings infested with aphids were treated with 5 mM DMTU (an H_2_O_2_ scavenger). The DAB staining results shown in Fig. 11 demonstrated that DMTU treatment inhibited the *S. graminum* feeding-induced production of H_2_O_2_ in wheat leaves and the symptoms of damage in wheat leaves caused by *S. graminum* feeding. The delayed fluorescence and chlorophyll content were also assessed, and the results showed that the DMTU-treated infested leaves showed decreased chlorophyll degradation and that the chlorophyll content in the DMTU-treated leaves was significantly higher than that in the non-DMTU-treated leaves infested with *S. graminum* (F_2, 6_ = 13.93, *P* = 0.0056).

## Discussion

A previous study showed that *S. graminum* feeding led to obvious feeding damage and the loss of chlorophyll in aphid-susceptible winter wheat accession Beijing 837 [18]. Similarly, serious chlorosis symptoms were observed on another winter wheat accession, Zhongmai 175, after 48 h of *S. graminum* feeding in this study, and this effect was also accompanied by a significant reduction in the total chlorophyll content of the wheat leaves, further demonstrating the phytotoxic effects of *S. graminum* on susceptible wheat plants. To further compare the similarities and differences between the responses to *S. graminum* and *S. avenae* feeding at the molecular level, a comparative transcriptome analysis of wheat leaves after aphid feeding was performed. We found that more than 20,000 genes were significantly regulated in wheat infested with *S. graminum*, but only 1700 genes were significantly modulated after 48 h of *S. avenae* feeding, which indicated that the physiological changes induced by *S. graminum* are notably different from those induced by *S. avenae* and that various metabolic pathways are involved in the development of damage caused by *S. graminum* feeding. Moreover, many genes involved in plant photosynthesis were strongly downregulated after *S. graminum* feeding, and this finding provides molecular evidence showing that chlorosis is induced by *S. graminum*.

### *S. graminum* feeding induces stronger plant defence responses than *S. avenae*

Piercing-sucking hemipteran insects, such as aphids and whiteflies, mainly induce SA-mediated defence signal pathways [28, 29]. However, some studies have also demonstrated that genes involved in both the JA and SA defence response pathways, such as *LOX*, *PIs*, *PAL*, and *PR1*, are significantly upregulated in response to aphid feeding [20, 30, 31]. Similarly, we found that both *S. avenae* and *S. graminum* feeding significantly increased the expression levels of genes related to the SA, JA and ET signalling pathways. Plant defence responses activated by aphids are closely associated with the plant species, aphid density and infestation time [32, 33]. In the future, we will further identify the wheat defence responses induced by *S. graminum* and *S. avenae* feeding under various aphid densities and feeding periods. Although both of these cereal aphids induced both SA-, JA- and ET-dependent defence pathways, *S. graminum* feeding induced the expression of more genes involved in plant defence pathways in this study. For example, 30 *PAL* genes were upregulated in response to *S. graminum* feeding, but only four *PAL* genes were upregulated in response to *S. avenae* feeding. The transcript levels of five ET-responsive genes were upregulated in response to *S. graminum,* but only one was modulated in response to *S. avenae* feeding. Zhang et al. demonstrated that the fold changes in the expression levels of *PR* genes and the SA contents in wheat leaves were significantly greater after *S. graminum* feeding than after *S. avenae* feeding [22]. Argandoña et al. also suggested that *S. graminum* induced more ethylene production than the non-phytotoxic aphid *Rhopalosiphum padi* [34]. The stronger defence responses activated by *S. graminum* feeding than by *S. avenae* feeding might be responsible for the induction of chlorosis in wheat.

### Genes encoding plant cell-modifying proteins are significantly upregulated in response to *S. graminum* feeding

Multiple modifications can be triggered in cell walls in response to microbial and insect attack [35]. Callose deposition in crop plants is observed in response to biotrophic fungal infection at papillae sites and in sieve elements in response to aphids [36, 37]. It has been proposed that callose deposition impedes fungal attacks at the sites of attempted penetration in epidermal cells and thereby supports pathogen resistance [38]. The transcript levels of callose synthases were significantly upregulated in response to *S. graminum* and *S. avenae* feeding. Obvious callose accumulation was also observed in wheat leaves after *S. avenae* and *S. graminum* feeding. However, the role of callose in aphid-plant interactions remains unknown. It has been hypothesized that callose deposition induced by aphids is involved in the sealing of the sieve pores as a phloem defence mechanism that impedes mass flow and prevents the flow of nutrients to piercing-sucking insects [39].

Many studies have demonstrated that the damage symptoms induced by pathogens and herbivores in plants are caused by the secretion of plant cell wall enzymes during the process of pathogen infection and mirid *Lygus hesperus* feeding [40, 41]. Additionally, the activities of PEMs and PGs have also been detected in *S. graminum* watery saliva, and the injection of these commercial enzymes in plant leaves causes damage symptoms similar to those induced by *S. graminum* feeding [42, 43]. However, pectinase activity has also been detected in the saliva of non-phytotoxic aphids, such as *S. avenae* and *A. pisum* [43, 44]. Transmission electron microscopy has shown that stylets predominantly penetrate between the layers of cellulose fibres and not via the middle lamella pectin layer [45]. The role of the saliva pectinases of *S. graminum* in the induction of chlorosis remains unclear. Interestingly, in our study, many plant-derived enzymes and proteins involved in plant cell wall modifications, such as PGs, PEMs and expansins, were induced by *S. graminum* feeding, but none were induced by *S. avenae* feeding. The upregulation of PG and PEM activity might result in the degradation of the cell wall around aphid feeding sites. Plant cells exploit complicated mechanisms for sensing the loss of cell wall integrity (CWI) during biotic stress and activate a variety of defence responses [46]. For instance, the production of oligogalacturonic acid (OGA) fragments derived from the degradation of plant cell walls has been shown to trigger oxidative bursts, hypersensitive responses (HR), and other downstream defence responses in many plant species as a host-derived DAMP [47, 48], and these effects might further promote the induction of damage symptoms. In addition, ethylene production has been shown to be involved in the induction of plant cell wall-modifying proteins and the death of plant tissues. The role of PCMDPs and the ethylene pathway in the induction of chlorosis symptoms by *S. graminum* needs to be further investigated.

### *S. graminum* feeding induces strong ROS-scavenging activity in wheat leaves

Aphid feeding usually leads to oxidative stress in host plants [49]. Oxidative stress is controlled by cellular antioxidant mechanisms in which multiple enzymatic scavengers, such as POD, APx, and CAT, are utilized by the cell to limit damage from reactive oxygen species [50, 51]. The transcriptomic and enzymatic results showed that both *S. graminum* and *S. avenae* feeding increased the transcript levels and enzyme activities of ROS scavengers in wheat leaves. However, the expression levels and activity of antioxidants, particularly POD, induced by *S. graminum* feeding were notably higher than those induced by *S. avenae*, which suggested that *S. graminum* infestation results in strong oxidative stress and substantial H_2_O_2_ accumulation. Although ROS scavengers were significantly upregulated in response to *S. graminum* infestation, ROS production can exceed the cellular antioxidant capacity, resulting in oxidative damage to cellular components and cell death in leaves.

### Induction of high H_2_O_2_ accumulation by *S. graminum* feeding is involved in leaf chlorosis

H_2_O_2_ is involved in the activation of HR, which is characterized by the rapid death of cells in the region surrounding the site of pathogen infection site [52, 53]. To further investigate the roles of H_2_O_2_ in the induction of feeding damage caused by *S. graminum* feeding, the accumulation of H_2_O_2_ in wheat leaves was detected. We found that *S. graminum* feeding induced the obvious accumulation of H_2_O_2_ at feeding sites, but *S. avenae* feeding had no significant effects on H_2_O_2_ production*.* In addition, seedlings treated with the H_2_O_2_ scavenger DMTU showed reductions in the chlorosis symptoms and chlorophyll loss triggered by *S. graminum* feeding. These results demonstrate that H_2_O_2_ accumulation plays important roles in the induction of chlorosis in wheat leaves in response to *S. graminum* feeding.

Aphid saliva is known to be involved in the induction of plant defence responses, and the eliciting activity of watery saliva of other aphid species such as *M. persicae* and *S. avenae* has been investigated [54, 55]. The transient overexpression of Mp10, a salivary protein of *M. persicae,* induces plant defence responses and obvious chlorosis in *N. benthamiana* [56]. Specific elicitors or pathogen-like toxins in *S. graminum* saliva are likely involved in the induction of chlorosis. Comparative analyses of the salivary proteomes of four differentially virulent *S. graminum* biotypes revealed six salivary proteins with significant proteomic variation, and these proteins might thus be involved in the induction of feeding damage in plants [57]. Further research is required to identify the virulence factors in the salivary proteins of *S. graminum* and the mechanism underlying the induction of chlorosis.

## Conclusions

In summary, the transcriptomic profiling of wheat performed in this study revealed similarities and differences among the responses of wheat to feeding by the phytotoxic aphid *S. graminum* and the non-phytotoxic aphid *S. avenae*. Both aphids induced the JA, SA and ET signalling pathways, but *S. graminum* triggered stronger plant defence responses *and greater ROS*-*scavenging activity* than *S. avenae*. A cytological analysis showed that aphid feeding induced callose deposition in wheat leaves and that substantial H_2_O_2_ accumulation was induced by *S. graminum* feeding. Our results also demonstrated that H_2_O_2_ plays vital roles in the induction of chlorosis in wheat leaves in responses to *S. graminum* feeding. Our future studies will focus on the mechanisms of H_2_O_2_ accumulation induced by *S. graminum* feeding and the roles of salivary proteins of *S. graminum* in the induction of chlorosis symptoms in wheat.

## Methods

### Plants and aphids

Seeds of *Triticum aestivum* var. Zhongmai 175 were germinated in distilled water for 3–4 days at a temperature of 25 ± 1 °C in a Petri dish. Healthy seedlings of similar sizes were planted in 7.2 × 7.2 cm plastic plots filled with organic soil and grown under controlled environmental conditions in climate chambers with a temperature of 20 ± 1 °C, a 40–60% relative humidity and a 14-h-light/10-h-dark photoperiod. Clones of *S. graminum* and *S. avenae* were maintained on the wheat plants (Zhongmai 175) as described previously [18].

### Aphid infestation

At the two-leaf stage (12-day old plants), 20 apterous adult *S. graminum* or *S. avenae* were confined on the first leaf of wheat seedlings using a clip cage as described previously [18]. New-born nymphs produced by aphid adults were carefully removed every 12 h using a brush. After 48 h of feeding, all the aphids were removed, and leaf tissues of approximately 2.5 × 2.5 cm from the aphid feeding sites of each plant were harvested flash frozen with liquid nitrogen and stored at − 80 °C until further processing for RNA extraction. Detection of delayed fluorescence and histological staining were conducted immediately after sample collection. Three leaf sections covering the aphid feeding sites were collected from three independent plants and pooled to form one biological replicate. Three biological replicates were performed for each treatment.

### Changes in chlorophyll levels in wheat leaves after aphid infestation

Delayed fluorescence is associated with extremely weak light emitted by chlorophyll molecules in plants and can reflect the chlorophyll content, providing a powerful tool for studying stress reactions in plants. The chlorophyll content in wheat leaves were each infested by 20 aphids as described above was first detected using the NightShade LB 985 In vivo Plant Imaging System (Berthold Technologies, Bad Wildbad, Germany). After 48 h of aphid feeding, the leaves were cut and immediately illuminated for 30 s with an LED panel. After the light was switched off, the delayed fluorescence was measured immediately using the NightShade system. The exposure time was set to 30 s using 4-by-4 pixel binning. The total chlorophyll content in wheat leaves after aphid infestation was also examined using a Chlorophyll Assay Kit (Solarbio, Beijing, China) according to the manufacturer’s instructions. In brief, 0.1 g of fresh leaf tissues was ground to a fine powder and extracted with 2 mL of 80% acetone (v/v) at 4 °C for overnight. The homogenate was centrifuged at 4000 g for 10 min at 4 °C, and the supernatant was used for the chlorophyll assay. The amounts of chlorophyll were detected spectrophotometrically, by reading the absorbance at 645 and 663 nm (DU800, Beckman, USA), and then calculated as described previously [58].

### RNA preparation and sequencing library construction

Wheat leaves were first infested with aphids for 48 h as described above. The total RNA from the wheat leaves was extracted with the TRIzol reagent (Invitrogen) according to the manufacturer’s recommended protocol. The RNA concentration was measured using a NanoDrop 2000 spectrophotometer (Thermo Fisher Scientific, Inc., USA), and the RNA integrity was evaluated using an Agilent 2100 Bioanalyzer (Agilent Technologies, Santa Clara, CA, USA). Samples with an RNA integrity number (RIN) ≥ 7.0 were used in the subsequent analysis. Libraries were constructed using the TruSeq Stranded mRNA LT Sample Prep Kit (Illumina, San Diego, CA, USA) according to the manufacturer’s instructions and were sequenced on the Illumina sequencing platform (Illumina HiSeq 4000), which generated 150-bp paired-end reads were generated.

### RNA sequencing and data analysis

The raw data (raw reads) were filtered to obtain high-quality reads by removing the reads containing adaptor sequences, more than 3% ambiguous bases (noted as N) or more than 50% low-quality bases (Phred quality score Q < 30). The resulting high-quality clean reads were then mapped to the reference genome (https://plants.ensembl.org/Triticum_aestivum/Info/Index) using TopHat2 with the default values [59]. The fragments per kilobase of exon model per million mapped reads (FPKM) data were used to estimate the transcript expression levels in all the samples, and the genes with more than 1 FPKM in at least one sample of wheat leaves were used for further analysis [60]. The differentially expressed genes (DEGs) between the control and treated samples were screened using DESeq based on the following criteria: adjusted *p* value (*q* value) threshold < 0.00001 and |log_2_ FC| ≥ 1. A PCA of nine different leaf samples was performed based on pairwise comparisons using the DESeq package of R [61]. GO enrichment and KEGG pathway enrichment analyses of the DEGs were performed using R based on the hypergeometric distribution [62]. To annotate the functions of the transcripts, the unigenes were blasted against the Nr database using the BLAST programme with an *E*-value ≤1e-5.

### Assays of antioxidant enzymes in wheat leaves after aphid infestation

The leaves were infested with 20 apterous adult *S. graminum* or *S. avenae* as described previously. The activities of peroxidase (POD), superoxide dismutase (SOD), catalase (CAT) and ascorbate peroxidase (APx) in the wheat leaves at different time points after the aphid infestation (12 h, 24 h, 48 h and 72 h) were examined using corresponding kits (Jiancheng Bioengineering Institute, Nanjing, China) according to the manufacturer’s instructions. Briefly, 0.2 g of fresh leaf tissues was ground with 1.5 mL of ice-cooled 50 mM Na-phosphate buffer (pH = 7.8) containing 0.1 mM EDTA and 1.0% (w/v) polyvinylpyrrolidone. The homogenate was centrifuged at 15,000 g for 30 min at 4 °C, and the supernatant was immediately collected for further enzyme assays. The activities of POD, SOD, CAT, and APx were measured by following the changes in absorbance at 470 nm, 560 nm, 240 nm and 290 nm, respectively, according to a previous study [63].

### Detection of H_2_O_2_ and callose accumulation in wheat leaves after aphid infestation

The detection of H_2_O_2_ in wheat leaves by 3′-diaminobenzidine (DAB) staining was performed according to the histochemical methods described by Wang et al. [64] with some modifications. In brief, leaf segments previously infested with 20 apterous adults of *S. avenae* or *S. graminum* were immersed in 1 mg mL^− 1^ DAB solution (10 mmol L^− 1^ Na_2_HPO_4_, pH 3.8), and incubated in the dark overnight at room temperature. Then, the leaves were decolorized in boiling 95% ethanol for 10 min and hyalinized in saturated chloral hydrate. The stained leaves were imaged using an Olympus BX-63 microscope (Olympus Corporation, Japan). The endogenous H_2_O_2_ content in the wheat leaves after aphid feeding was determined using the protocols reported by Ferguson et al. [65]. For the visualization of callose, the leaves were first fixated, destained overnight in 1:3 acetic acid/ethanol (v/v) solution and washed in 150 mM K_2_HPO4 for 30 min. The leaves were subsequently incubated for 6 h with 150 mM K_2_HPO4 and 0.01% aniline blue for staining, and the callose depositions were observed and photographed with an Olympus SZX-16 fluorescence microscope (Olympus Corporation, Japan) using a DAPI filter.

### Wheat seedlings treated with DMTU solution

The leaves of wheat seedlings were treated with 5 mM dimethylthiourea (DMTU, a scavenger of H_2_O_2_) solution or deionized water (control) for 24 h and then infested with 20 apterous adults of *S. graminum* for 48 h. Assessments of DAB staining and delayed fluorescence analyses and an assessment of the chlorophyll content of the wheat leaves were then performed as described previously.

### Statistics analysis

All the data were analysed using SPSS Statistics 20.0 software (SPSS Inc., Chicago, IL., USA), and the differences between or among groups were examined through an independent-samples *t*-test or one-way analysis of variance (Duncan). *P* values less than 0.05 were considered statistically significant.

## Supplementary information

**Additional file 1: Table S1.** Summary of transcriptome data.

**Additional file 2: Data S1.** List of all DEGs after infestation by aphids in comparison with controls.

**Additional file 3: Figure S1.** KEGG enrichment.

**Additional file 4: Data S2.** DEGs involved in SA, JA and ET- dependent defence pathways in response to aphid feeding.

**Additional file 5: Data S3.** Transcript levels of genes involved in ROS scavenge after aphid feeding.

## Data Availability

The datasets used and analyzed during the current study are available from the corresponding author on reasonable request.

## Abbreviations

ACO: 1-aminocyclopropane-1-carboxylate oxidase 1
ACS: 1-aminocyclopropane-1-carboxylate synthase
AOC: Allene oxide cyclase
AOS: Allene oxide synthase
APx: Ascorbate peroxidase
BYDV: Barley yellow dwarf virus
CAT: Catalase
CWI: Cell wall integrity
DAB: 3′-diaminobenzidine
DAMPs: Damage associated molecular patterns
DEGs: Differentially expressed genes
DMTU: Dimethylthiourea
ET: Ethylene
EXP: Beta-expansin
FDR: Adjusted *p* value
FPKM: Fragments per kilobase of exon model per million mapped reads
GPx: Glutathione peroxidase
H_2_O_2_: Hydrogen peroxide
HAMPs: Herbivory associated molecular patterns
HR: Hypersensitive response
JA: Jasmonic acid
LOX: Lipoxygenase
MeJA: Methyl jasmonate
OGA: Oligogalacturonic acid
PAE: Pectin acetylesterase
PAL: Phenylalanine ammonia-lyase
PEM: Pectinesterase
PG: Polygalacturonase
PIs: Proteinase inhibitors
POD: Peroxidase
PR protein: Pathogenesis-related protein
RNA-Seq: High-throughput RNA sequencing
ROS: Reactive oxygen species
RuBisCO: Ribulose bisphosphate carboxylase oxygenase
SA: Salicylic acid
SEs: Sieve elements
SOD: Superoxide dismutase
