# Supplementary figures and images for: Comparative transcriptome and histological analyses of wheat in response to phytotoxic aphid Schizaphis graminum and non-phytotoxic aphid Sitobion avenae feeding
Source: BMC Plant Biol. 2019 Dec 10;19:547. doi: 10.1186/s12870-019-2148-5 (PMC6902339; doi:10.1186/s12870-019-2148-5)

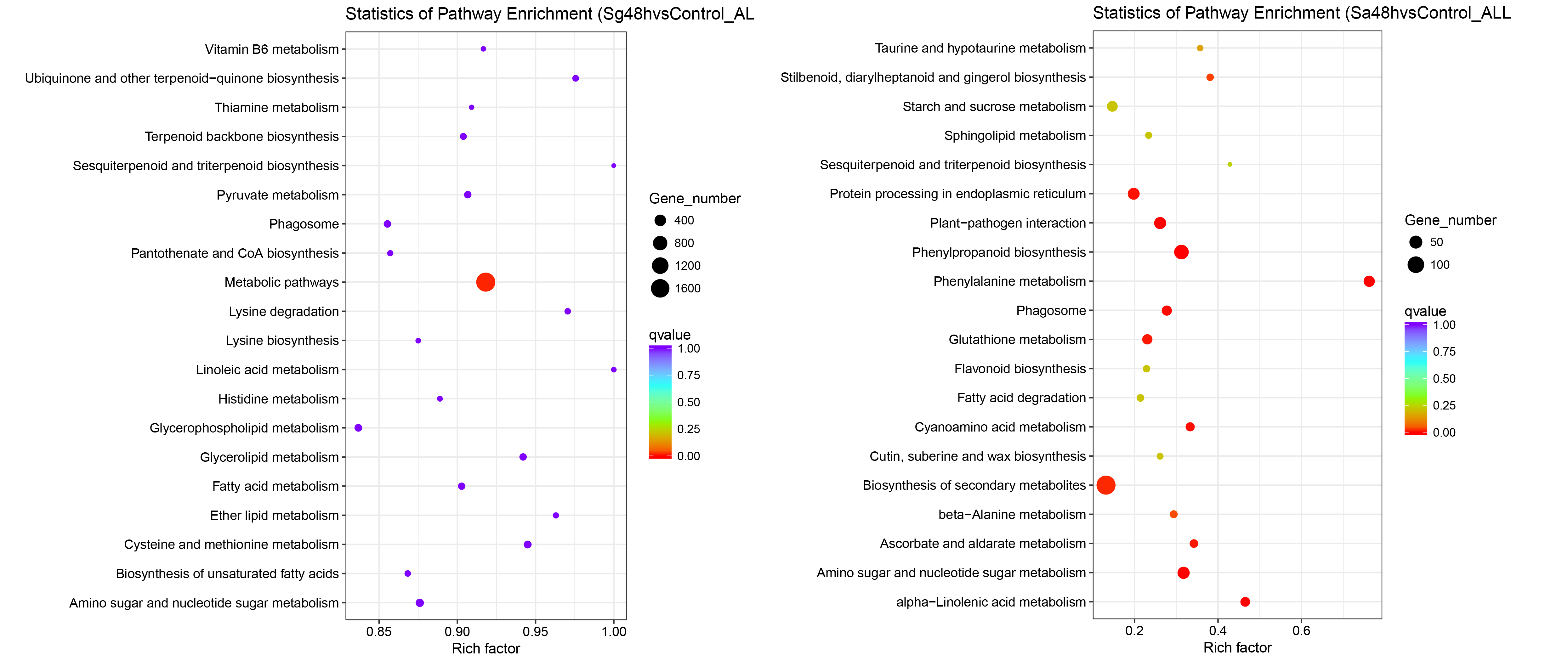

Supplement: Supplementary file 3 — Additional file 3: Figure S1. KEGG enrichment. [file 12870_2019_2148_MOESM3_ESM.tif]
